# *Hyalomma* spp. ticks and associated *Anaplasma* spp. and *Ehrlichia* spp. on the Iran-Pakistan border

**DOI:** 10.1186/s13071-021-04956-3

**Published:** 2021-09-14

**Authors:** Nayyereh Choubdar, Fateh Karimian, Mona Koosha, Jalil Nejati, Mohammad Ali Oshaghi

**Affiliations:** 1 Department of Medical Entomology and Vector Control, School of Public Health, Tehran University of Medical Sciences, Tehran, Iran; 2 Department of Parasitology, Pasteur Institute of Iran, Tehran, Iran; 3 Department of Public Health, School of Public Health, Zahedan University of Medical Sciences, Zahedan, Iran

**Keywords:** Anaplasmosis, Ehrlichiosis, Hard ticks, Iran, Pakistan, Tick-borne diseases

## Abstract

**Background:**

Anaplasmosis and ehrlichiosis are tick-borne diseases affecting humans and livestock, particularly in tropical and subtropical regions. Animal husbandry is the main activity of people on the borders of Iran and Pakistan, with thousands of cattle crossing the border each week.

**Methods:**

PCR and sequencing of the *16S rRNA* gene was used to determine the percentage and geographical distribution of the pathogens carried by *Hyalomma* spp. (*n* = 306) collected from 126 goats, cattle and camels in the region between November 2017 and late March 2018.

**Results:**

In total, 1124 hard ticks including 1020 *Hyalomma* spp. ticks belonging to six species (*Hyalomma anatolicum*, *Hyalomma asiaticum*, *Hyalomma marginatum*, *Hyalomma dromedarii*, *Hyalomma schulzei,* and *Hyalomma detritum*) were found on the borders of Iran and Pakistan, with *H. anatolicum* being the most prevalent tick species. *Anaplasma* spp. and/or *Ehrlichia* spp. DNA was found in 68.3% of the engorged tick specimens (*n* = 256). Sequencing of a subset (12.6%) of PCR-positive samples revealed *Anaplasma ovis*, *Anaplasma marginale*, and *Ehrlichia ewingii* DNA in 81.8%, 9.1%, and 9.1% of the ticks, respectively. To our knowledge, this is the first report of *E. ewingii*, an important human pathogen, in Iran.

**Conclusions:**

Based on molecular analysis, three pathogenic Anaplasmataceae were detected in six *Hyalomma* spp. parasitizing cattle, goats and camels, confirming the presence of these pathogens along the Iran-Pakistan border.

**Graphical Abstract:**

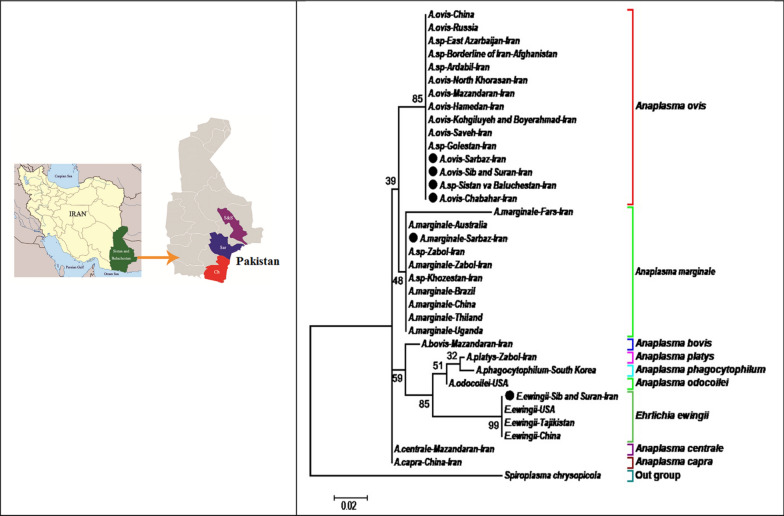

**Supplementary Information:**

The online version contains supplementary material available at 10.1186/s13071-021-04956-3.

## Background

Ticks are considered to be the second most common vector of human diseases worldwide after mosquitoes, but they are the most important vectors of disease-causing pathogens in domestic and wild animals. Indeed, ticks transmit a wide variety of pathogens, including viruses, bacteria and protozoa, to their vertebrate hosts [1].

For instance, anaplasmosis is caused by a number of established and emerging tick-borne pathogens, including *Anaplasma phagocytophilum*, *Anaplasma marginale*, *Anaplasma ovis*, *Anaplasma centrale*, *Anaplasma bovis*, *Anaplasma capra*, *Anaplasma platys*, and ‘*Candidatus* Anaplasma camelii’, which are found worldwide, particularly in tropical and subtropical regions, including Iran [2–6]. *Anaplasma* spp. may infect humans and a broad range of wild and domestic mammals, including horses, dogs, cats, deer, goats, sheep, cattle, camel, and other ruminants [4, 6, 7]. Ehrlichiosis caused by *Ehrlichia* spp. is another tick-borne disease [8], and some *Ehrlichia* spp. have been identified as pathogens in humans. For example, *Ehrlichia chaffeensis* causes human monocytic ehrlichiosis and *Ehrlichia ewingii* is an agent of human granulocytic ehrlichiosis [9]. The zoonotic nature of the human ehrlichiosis is supported by reports of natural infections with the same *Ehrlichia* spp. in dogs, deer, horses, and rodents [10]. Granulocytic ehrlichiosis in humans has been described in immunocompromised and immunocompetent patients, causing headache, fever, myalgia, vomiting, nausea, acute renal failure, thrombocytopenia, leukopenia and increased liver enzyme activities [11–13].

Iran has a variety of climates: a mild cold climate in the high mountains; continental and arid climates in the plateau; a mild and damp climate on the Caspian coast; and a hot desert climate on the southern coast and in the south-east. Each climate may provide conditions suitable for the development of different tick species, which probably explains the difference in the epidemiology of anaplasmosis and ehrlichiosis in different regions [14]. Sistan and Baluchistan Province in the south-east corner of Iran has a long border with Pakistan and Afghanistan, where infectious diseases do not respect international boundaries [15]. Animal husbandry is one of the main activities of the people in the province, and every week thousands of head of livestock, including sheep, goats, cows, camels, and buffaloes, cross the borders between the countries [16].

Although *Anaplasma* spp. and *Ehrlichia* spp. have been identified by molecular assays in livestock in Iran, their presence in their vectors has been much less studied. There have been only a few studies on the detection of *Anaplasma* spp. and *Ehrlichia* spp. in ticks in Iran, which reported the presence of infected ticks in the north and other regions of Iran [14, 17, 18].

Ixodid ticks play an important role in maintaining *Anaplasma* spp. and *Ehrlichia* spp. in nature [19, 20]. Nonetheless, while *Hyalomma* spp. have been suggested as vectors of *A. marginale* [21], there has been little research on their possible involvement in *Anaplasma* spp. and *Ehrlichia* spp. transmission. Here, we report the occurrence of *Hyalomma* spp. infesting various domestic animals in the Iranian Province of Sistan and Baluchistan, with notes on *Anaplasma* spp. and *Ehrlichia* spp. infections in these ticks.

## Methods

### Sample collection and tick identification

The regions investigated include three districts of Sib & Suran County (Hiduj district; geographical coordinates 27°00′02″N, 62°07′01″E), Sarbaz County (Pishin district; geographical coordinates 30°35′5.31″N, 66°59′41.19″E) and Chabahar County (Negour district; geographical coordinates 25°23′20.84″N, 61°8′18.96″E), all of which are located in the south-east of Iran, close to the border with Pakistan. The collection of ticks was performed in three randomly selected major animal husbandry farms in each district, between November 2017 and late March 2018, i.e. at the season when adult ticks are most active in the region. In total, 1124 ticks were collected from goats (*n* = 80), cattle (*n* = 34), and camels (*n* = 12). Tick collection was arbitrarily conducted, depending upon the availability of the domestic animals for a 15-min examination per animal, but efforts were made to obtain a widespread representative sample from the different animal species included in the study. All ticks were transferred to vials and labelled according to their geographical origin and the animal from which they were obtained. The collected ticks were subsequently transferred to the Entomology Laboratory in the School of Public Health at the Tehran University of Medical Sciences and were identified to species level, based on microscopic observation of external morphological characteristics according to the identification keys [22–24].

### DNA extraction

After species identification, the ticks were sterilized by immersion in 70% alcohol, washed in distilled water, dried on filter paper in a laminar-flow hood, and then stored at −80 °C until the DNA extraction.

Individual ticks were frozen in liquid nitrogen and then ground in an Eppendorf microtube. The DNA was then extracted using the G-spin Genomic DNA Extraction Kit (iNtRON Biotechnology, South Korea), according to the manufacturer’s instructions. The precipitated DNA samples were quantified using a spectrophotometer (Thermo Scientific™ NanoDrop™ One, Wilmington, DE, USA), and their integrity was assessed using 1% agarose gel electrophoresis. Then, the extracted DNA was suspended in sterile distilled water and stored at −20 °C. Of the ticks collected, all the engorged ticks were analysed, and a subset (*n* = 50) of the unfed ticks were also tested for the presence of *Anaplasma* spp. and *Ehrlichia* spp.

### Molecular detection of *Anaplasma* spp. and *Ehrlichia* spp. in ticks

The *16S rRNA* gene of *Anaplasma* spp. and *Ehrlichia* spp. was amplified using the nested PCR protocol designed by Rar et al*.* [25]. The forward and reverse primers for initial reactions were Ehr1 (5′-GAA CGA ACG CTG GCG GCA AGC-3′) and Ehr2 (5′-AGT A(T/C)C G(A/G)A CCA GAT AGC CGC-5′) and for nested reactions were Ehr3 (5′-TGC ATA GGA ATC TAC CTA GTA G-3′) and Ehr4 (5′-CTA GGA ATT CCG CTA TCC TCT-3′). The size of final PCR products was 524 base pairs (bp). PCR reactions were performed in a 25 μl reaction mixture, containing 12.5 μl of the Hot Start Taq 2 $\times$ Master Mix, 1 μl of each of the forward and reverse primers, 2 μl of DNA template and 7.5 μl of nuclease-free water to bring the volume to 25 μl. PCR reactions were performed in a DNA Mastercycler Personal PCR machine (Eppendorf, Germany) and PCR conditions were: 15 min at 95 °C for initial denaturation, then 60 s at 94 °C for denaturing, 60 s at 57 °C for annealing, and 60 s at 72 °C for extension for 35 cycles, and then a final extension for 10 min at 72 °C. The products (2 μl) of the first PCR were used as the template for the second PCR, which was carried out under the same conditions and reaction mixture as the first, except that nesting primers were used [25]. Two negative controls (one of double-distilled water and one of an un-infected tick DNA template) and a positive control (a confirmed sequenced *A. bovis* DNA isolated from a tick) were included in each PCR run. To ensure the presence of viable DNA, the barcoding region of the tick *COI* gene was used as endogenous control [26].

To assess the presence of expected bands for *Anaplasma* spp. and *Ehrlichia* spp., the PCR products were electrophoresed in a 1.5% agarose gel, and the size of each PCR product was estimated using a 100 bp ladder and visualized with a UV transilluminator.

### DNA sequencing and phylogenetic analysis

The positive PCR products were purified, and bidirectional DNA sequencing was performed by the Sanger method, using the same inner PCR primers that were used for nested PCR amplification. The sequences obtained were edited and assembled using Chromas (http://www.technelysium.com.au/chromas.html) and BioEdit [27] software to construct consensus sequences, which were then analysed using the NCBI Blast database (Nucleotide collection) (https://www.ncbi.nlm.nih.gov/). The consensus high-confidence sequences were aligned with other sequences that were available in GenBank, using multiple sequence alignments available in CLUSTAL Omega (https://www.ebi.ac.uk/Tools/msa/clustalo). For phylogenetic analysis, the representative sequences of *Anaplasma* spp. and *Ehrlichia* spp. obtained in this study were combined with a subset of available representative sequences of all *Anaplasma* spp. and *E. ewingii*, using *Spiroplasma* sp. sequences as an outgroup [28-31]. Details of these sequences are shown as supplementary data (Additional file 1: Table S1). All the DNA sequences used for alignment were cut to obtain a consistent region (470 bp), and phylogenetic analyses were performed using the MEGA 7 software [32]. The data were aligned and the maximum likelihood method was employed to construct a phylogenetic tree. The same program was utilized to evaluate the stability of the obtained tree through bootstrap analysis with 1000 replicates.

## Results

### Tick species and abundance

A total of 1124 hard ticks were collected which were classified into three genera: *Hyalomma* (*n* = 1020, 90.7%), *Rhipicephalus* (*n* = 68, 6.1%), and *Dermacentor* (*n* = 36, 3.2%). All of the 1020 ticks collected were morphologically identified as belonging to one of six *Hyalomma* spp., with *Hyalomma anatolicum* being the most common species in all three districts. In detail, the tick species were found to be *H. anatolicum* (*n* = 462; 228 from cattle and 234 from goats), *Hyalomma asiaticum* (*n* = 143; 87 from camels, 25 from goats, and 31 from cattle), *Hyalomma marginatum* (*n* = 203; 134 from cattle, 66 from goats and three from camels), *Hyalomma dromedarii* (*n* = 188; 46 from goats and 142 from camels), *Hyalomma schulzei* (*n* = 17 from goats), and *Hyalomma detritum* (*n* = 6 from camels) (Table 1). The average number of ticks on camels, cattle, and goats were 19.8, 11.6, and 4.9, respectively. The collected tick specimens were mix of unfed (75%), partially fed (15.1%), and fully engorged (9.9%). Unfed were very small, often black or brown colour, partially engorged (partially swollen), which means the tick has had a partial blood meal, and engorged (swollen, usually blue-grey in colour) (Table 1).

**Table. 1:** Details of *Hyalomma* spp. specimens collected from animals in Sistan and Baluchistan Province, south-east corner of Iran, 2017–2018

Location	No. of animals examined			Tick species	No. of ticks on animal			Subtotal	Male/female	U/PE/FE	Total
	Ct	Go	Cm		Ct	Go	Cm				
Chabahar	14	29	1	*Hyalomma marginatum*	41	36	0	77	19**/**58	53**/**16**/**8	312
				*Hyalomma anatolicum*	134	59	0	193	47**/**146	149**/**24**/**20	
				*Hyalomma asiaticum*	17	25	0	42	11**/**31	30**/**8**/**4	
Sarbaz	13	23	4	*Hyalomma marginatum*	19	13	0	32	8/24	26/2/4	322
				*Hyalomma anatolicum*	43	132	0	175	44/131	126/31/18	
				*Hyalomma dromedarii*	0	46	52	98	24/74	80/7/11	
				*Hyalomma schulzei*	0	17	0	17	5/12	12/3/2	
Sib and Suran	7	28	7	*Hyalomma asiaticum*	14	0	87	101	25/76	79/14/8	386
				*Hyalomma dromedarii*	0	0	90	90	22/68	64/12/14	
				*Hyalomma detritum*	0	0	6	6	2/4	4/0/2	
				*Hyalomma marginatum*	75	17	3	95	24/71	74/21/0	
				*Hyalomma anatolicum*	51	43	0	94	24/70	68/16/10	
Total	34	80	12		394	388	238	1020	255**/**765	765**/**154**/**101	1020

Ct, cattle; Go, goat; Cm, camel; U, unfed; PE, partially engorged; FE, fully engorged

### *Anaplasma* spp. and *Ehrlichia* spp. infections in ticks

Using generic EHR primers, a subset (*n* = 50) of non-engorged tick specimens, and all of the 255 fully or partially engorged tick specimens, comprising 63 males (25%) and 192 females (75%), were tested for the presence of *Anaplasma* spp. and *Ehrlichia* spp. The tested specimens were representatives of the ticks collected from different animals in the three regions. The positive results were obtained with 68.6% (175 out of 255) of the engorged specimens, whereas non-engorged specimens were all negative. The species, number, and percentage of pathogen infection in *Hyalomma* spp. ticks at each collection site are shown in Table 2. A sub-set of positive PCR amplicons was sequenced and the consensus sequences were deposited in GenBank (accession numbers shown in Table 3). Details of the *Anaplasma* spp. and *Ehrlichia* spp.-positive samples are provided in Table 3.

**Table. 2:** Details of Anaplasmataceae infections in different *Hyalomma* spp. collected from Sistan and Baluchistan Province, south-east corner of Iran, 2017–2018

Tick species	No. of collected samples	No. of specimens tested (%)	Anaplasmataceae positive (%)	No. of specimens sequenced
*Hyalomma anatolicum*	462	114 (24.7)	78 (67.8)	7
*Hyalomma asiaticum*	143	35 (24.4)	24 (68.5)	3
*Hyalomma dromedarii*	188	47 (25)	32 (68.1)	4
*Hyalomma marginatum*	204	52 (25.4)	36 (69.2)	4
*Hyalomma detritum*	6	2 (33.3)	2 (100)	2
*Hyalomma schulzei*	17	5 (29.4)	2 (40)	2
Total	1020	255 (25.0)	175 (68.3)	22

**Table. 3:** Details of *Anaplasma* spp*.* and *Ehrlichia* spp. infections in *Hyalomma* spp. from three districts of Sistan and Baluchistan Province, south-east corner of Iran, 2017–2018

Tick species	Tick sex	Host	District	Pathogen species	GenBank ID number
*Hyalomma anatolicum*	Female	Cattle	Chabahar	*Anaplasma ovis*	MK310471
*Hyalomma anatolicum*	Female	Cattle	Sib and Suran	*Anaplasma ovis*	MK310472
*Hyalomma anatolicum*	Female	Cattle	Sib and Suran	*Anaplasma ovis*	MK310473
*Hyalomma anatolicum*	Male	Goat	Sib and Suran	*Anaplasma ovis*	MK310474
*Hyalomma anatolicum*	Female	Goat	Sarbaz	*Anaplasma ovis*	MK310475
*Hyalomma anatolicum*	Female	Goat	Sarbaz	*Anaplasma ovis*	MK310476
*Hyalomma asiaticum*	Female	Cattle	Sib and Suran	*Anaplasma ovis*	MK310477
*Hyalomma asiaticum*	Male	Goat	Sib and Suran	*Anaplasma ovis*	MK310478
*Hyalomma asiaticum*	Female	Cattle	Chabahar	*Anaplasma ovis*	MK310479
*Hyalomma dromedarii*	Female	Goat	Sarbaz	*Anaplasma ovis*	MK310480
*Hyalomma dromedarii*	Male	Camel	Sarbaz	*Anaplasma ovis*	MK310481
*Hyalomma marginatum*	Male	Cattle	Sib and Suran	*Anaplasma ovis*	MK310482
*Hyalomma marginatum*	Male	Goat	Chabahar	*Anaplasma ovis*	MK310483
*Hyalomma marginatum*	Female	Cattle	Chabahar	*Anaplasma ovis*	MK310484
*Hyalomma detritum*	Male	Camel	Sib and Suran	*Anaplasma ovis*	MK310485
*Hyalomma detritum*	Male	Camel	Sib and Suran	*Anaplasma ovis*	MK310486
*Hyalomma schulzei*	Female	Goat	Sarbaz	*Anaplasma marginale*	MK310487
*Hyalomma schulzei*	Male	Goat	Sarbaz	*Anaplasma marginale*	MK310488
*Hyalomma anatolicum*	Male	Cattle	Chabahar	*Anaplasma ovis*	MK310489
*Hyalomma dromedarii*	Female	Camel	Sib and Suran	*Ehrlichia ewingii*	MK310490
*Hyalomma dromedarii*	Male	Camel	Sib and Suran	*Ehrlichia ewingii*	MK310491
*Hyalomma marginatum*	Female	Cattle	Chabahar	*Anaplasma ovis*	MH480603

### Sequence and phylogenetic analysis

Analysis of the sequence data showed that the highest percentage of sequences obtained corresponded to *A. ovis* (18 out of 22, 81.8%). All of the strains of *A. ovis* detected in this study were identical, both to each other and to the other Iranian strains, and to the strains from China (GenBank: MG869525) and Russia (GenBank: KC484563). In addition to *A. ovis*, two *A. marginale* (9.1%) and two *E. ewingii* (9.1%) positive samples were detected. Sequences of *A. marginale* found in this study were identical to sequences from the USA, Brazil, Cuba, Iraq and China (GenBank: CP000030, CP023731, MK804764, MH551233, and KU586075, respectively). Similarly, the detected strains of *E. ewingii* obtained in this study were identical or highly similar to the isolates from Australia, the USA, Brazil, Thailand, and China (GenBank: NR_044747, NR_044747, HQ908082, NR_044747, and MN148615, respectively). The detected strains of *A. marginale* and or *E. ewingii* showed sequence similarities of 99–100% with sequences available in GenBank.

**Fig. 1:**
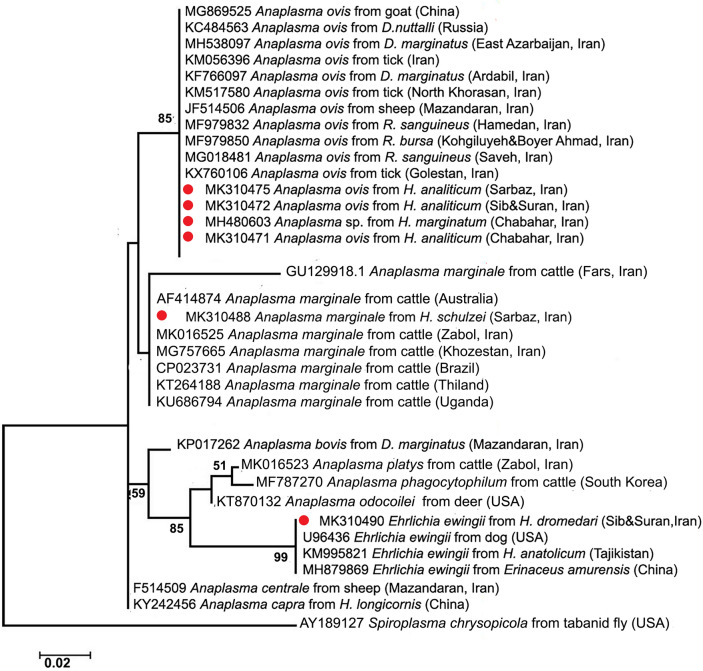
Phylogenetic relationships inferred from 470 bp of *16S rRNA* gene sequences of *Anaplasma* spp. and *Ehrlichia* spp. detected in this study and others available in GenBank. The representative sequences obtained in this study are marked with red circles. The tree was computed by maximum likelihood (MEGA 7.0 software). Bootstrap values (> 50%) are shown on nodes. For sequences from Iran, the county or province name is also provided

Phylogenetic analysis revealed that the *Anaplasma*/*Ehrlichia* spp. detected in this study clustered into four different clades, including (I) *A. ovis*, (II) *A. marginale*, (III) *A. platys-A. phagocytophilum-A. odocoilei*, and (IV) *A. centrale-A. capra* (Fig. 1). Both *E. ewingii* sequences obtained herein were associated with the branches of clade III.

## Discussion

This is the first comprehensive study of the *Hyalomma* spp. ticks from domestic animals and their associated *Anaplasma* spp. and *Ehrlichia* spp. along the Iran-Pakistan border. The results show that domestic animals in this area are infested with at least six *Hyalomma* spp., in addition to ticks belonging to other genera, which were not identified to species level herein. Moreover, at least two *Anaplasma* spp. and one *Ehrlichia* sp. were detected in the collected ticks. These results are generally in agreement with previous observations in Pakistan and in other parts of Iran [17, 33–36].

Herein, more than 90% of livestock-infesting adult ticks belonged to the genus *Hyalomma*, which agrees with the result of other researchers in Baluchistan of Pakistan where 525 out of 529 (99.2%) of the ticks belonged to this genus [37]. Among *Hyalomma* spp., *H. anatolicum* was the most common species and presented the widest geographical range. This three-host tick species was previously reported to be the most prevalent hard tick from different parts of Pakistan and Iran [17, 18, 33–35, 38–43].

The ecology of different *Hyalomma* spp. ticks may influence the risk of tick-borne diseases in the region [39, 41, 44–46]. The intense animal movements across Iranian regions and neighbouring countries may also facilitate the spread of these ticks. The infected ticks found in this study were feeding on the animals at the time of collection and were, therefore, potentially transmitting (or ingesting) *Anaplasma* spp. and *E. ewingii* while feeding. Therefore, the possibility that these ticks play a role in the transmission of these agents to domestic animals in the study area requires further investigation.

Except for *E. ewingii*, the pathogens found herein have previously been detected in ticks in several regions of Iran [14, 18, 33, 41–43]. However, we found the prevalence of *Hyalomma* spp. ticks carrying *Ehrlichia* spp. or *Anaplasma* spp. to be 68%, which is higher than previously reported from other parts of the country, including 4.6% in the south-eastern and north-western regions [33], 25% in the north [35], 43.84% in Meshkin-Shahr, Ardebil Province [43], and 55.5% in the four provinces of East Azerbaijan, Gilan, South Khorasan and Yazd [14]. These differences in tick infection rates could be related to several factors, including sampling methods, diagnostic methods, season, tick species, and feeding status of the ticks. Here, although the nested PCR technique used is known to be highly sensitive and specific, more nested PCR-positive samples should have been sequenced to determine the actual prevalence of *Anaplasma* spp. and *Ehrlichia* spp. infection in the ticks.

The results of this study, taken together with the above literature, have shown that different *Hyalomma* spp. could be involved in the transmission of pathogens to cattle, goats and camels in different regions of Iran. In addition to the *Hyalomma* spp. ticks, other ticks, including *Rhipicephalus bursa*, *Rhipicephalus sanguineus* sensu lato, *Dermacentor marginatus*, *Haemaphysalis erinacei*, and *Ixodes ricinus* have been reported as proven or putative vectors of different bacteria of the family Anaplasmataceae in Iran [17, 18, 33, 43].

The fact that no *Anaplasma* spp. or *Ehrlichia* spp. DNA was detected in the non-engorged specimens may suggest that there is little or no transovarial transmission of the bacteria in *Hyalomma* spp. ticks. Moore et al. [47] showed some evidence of transovarial transmission of *A. ovis* in *Dermacentor nuttalli*, although they did not detect any *A. ovis* DNA in larvae or nymphs. Another study [48] demonstrated the transovarial transmission of *A. marginale* in *Rhipicephalus microplus*, and that infected larvae can transmit the infection to susceptible hosts.

In this study, *A. ovis* was detected in five tick species: *H. anatolicum*, *H. asiaticum*, *H. marginatum*, *H. dromedarii*, and *H. detritum*, which were collected from cattle, goats, and camels. On the other hand, *A. marginale* and *E. ewingii* were found only in *H. schulzei* and *H. dromedarii* collected from goats and camels, respectively. Both *A. ovis* and *A. marginale* are important livestock pathogens [49], whereas *E. ewingii* is an important human pathogen [10, 50, 51] and is reported for the first time in Iran. However, the presence of *E. ewingii* should be confirmed by sequencing other genes (e.g., groEL), which was not possible in this study. *Anaplasma ovis* is distributed worldwide and is an important agent of anaplasmosis in small ruminants [52, 53]. For instance, bovine anaplasmosis is hyper-endemic in Sistan and Balouchestan Province, where 80% of goats have been shown to be infected with *A. ovis* [54]. Indeed, both *A. ovis* and *A. marginale* are established as the main agents of goat anaplasmosis in Iran [54].

In addition to *A. ovis* and *A. marginale*, other studies at the borders of Iran with Afghanistan and with Pakistan have reported the presence of different *Ehrlichia* spp. and *Anaplasma* spp. [18, 36, 40, 55] including *A. marginale*, *A. centrale*, *A. ovis*, *A. platys*-like organism, and *Ehrlichia minasensis*, and two uncharacterized species, namely, *Ehrlichia* sp. (Multan) and *Anaplasma* spp. (BL099-6) [18, 36, 40].

## Conclusions

In conclusion, we confirmed the presence of *A. ovis*, *A. marginale* and *E. ewingii* in *Hyalomma* spp. ticks collected from cattle, goats and camels on the Iran-Pakistan border. Further research is needed to confirm the role of these *Hyalomma* spp. and other ticks in the transmission of these pathogens in this region.

## Supplementary Information

**Additional file 1: Table S1.** Details of the bacterial species used for phylogenetic analysis in this study. *D*, *Dermacentor*, *Rh*, *Rhipicephalus*, *Hy*, *Hyalomma*, *Ha*, *Haemaphysalis. *

## Data Availability

All data generated or analysed during this study are included in this published article. The sequence data are available at NCBI database.

## Abbreviations

PCR: Polymerase chain reaction
Bp: Base pair
COI: Cytochrome oxidase subunit one
UV: Ultraviolet
NCBI: National Center for Biotechnology Information
