# Supplementary material for: Hyalomma spp. ticks and associated Anaplasma spp. and Ehrlichia spp. on the Iran-Pakistan border
Source: Parasit Vectors. 2021 Sep 14;14:469. doi: 10.1186/s13071-021-04956-3 (PMC8439021; doi:10.1186/s13071-021-04956-3)
Supplement: Supplementary file 1 — Additional file 1: Table S1. Details of the bacterial species used for phylogenetic analysis in this study. D, Dermacentor, Rh, Rhipicephalus, Hy, Hyalomma, Ha, Haemaphysalis. [file 13071_2021_4956_MOESM1_ESM.docx]

**Table S1.** Details of the bacterial species used for phylogenetic analysis in this study. *D*, *Dermacentor*, *Rh*, *Rhipicephalus, Hy, Hyalomma, Ha, Haemaphysalis.*

| Bacterial species | Host | Origin | GenBank  ID number | References |
| --- | --- | --- | --- | --- |
| 1. *ovis* | Goat  *D. nuttalli*  *D. marginatus*  Tick  *D. marginatus*  Tick  Sheep  *Rh. sanguineus*  *Rh. bursa*  *Rh. Sanguineus*  Tick  *Hy. anatolicum*  *Hy. anatolicum*  *Hy. marginatum*  *Hy. anatolicum* | China  Russia  Iran-East Azarbaijan  Iran-Borderline of Iran-Afghanistan  Iran- Ardabil  Iran-North Khorasan  Iran- Mazandaran  Iran- Hamedan  Iran- Kohgiluyeh  & Boyer-Ahmad  Iran-Saveh  Iran-Golestan  Iran- Sarbaz  Iran-Sib and Suran  Iran- Chabahar  Iran- Chabahar | MG869525  KC484563  MH538097  KM056396  KF766097  KM517580  JF514506  MF979832  MF979850  MG018481  KX760106  MK310475  MK310472  MH480603  MK310471 | [46]  Direct submission  Direct submission  Direct submission  [40]  Direct submission  [17]  Direct submission  Direct submission  Direct submission  Direct submission  This study  This study  This study  This study |
| *A. bovis* | *D. marginatus* | Iran- Mazandaran | KP017262 | Direct submission |
| 1. *marginale* | Cattle  *Hy. schulzei*  Cattle  Cattle  Cattle  Cattle  Beef cattle  Cattle | Australia  Iran-Sarbaz  Iran-Zabol  Iran-Khozestan  Brazil  China  Thiland  Uganda | AF414874  MK310488  MK016525  MG757665  CP023731  MF289480  KT264188  [KU686794](https://www.ncbi.nlm.nih.gov/nucleotide/KU686794.1?report=genbank&log$=nuclalign&blast_rank=8&RID=1BZU5K3101R) | [52]  This study  Direct submission  Direct submission  Direct submission  Direct submission  Direct submission  Direct submission |
| *A. platys* | Cattle | Iran- Zabol | MK016523 | Direct submission |
| *A. phagocytophilum* | Cattle | South Korea | MF787270 | Direct submission |
| *A. odocoilei* | Deer | USA | KT870132 | Direct submission |
| *A. centrale* | Sheep  Deer | Iran- Mazandaran  Japan | JF514509  AB588977 | Direct submission  Direct submission |
| *A. capra* [5] | *Ha. longicornis* | China | KY242456 | Direct submission |
| *E. ewingii* | *Hy. dromedari*  Dog  *Hy. anatolicum*  *Erinaceus amurensis* | Iran- Sib and Suran USA Tajikistan  China | MK310490  U96436  KM995821  MH879869 | This study  [53]  Direct submission Direct submission |
| *Spiroplasma chrysopicola* [54] | Tabanid flies | USA | AY189127 | [55] |
